# Web accessibility support for visually impaired users using link content analysis

**DOI:** 10.1186/2193-1801-2-116

**Published:** 2013-03-18

**Authors:** Hajime Iwata, Naofumi Kobayashi, Kenji Tachibana, Junko Shirogane, Yoshiaki Fukazawa

**Affiliations:** 1 Kanagawa Institute of Technology, 1030 Shimo-ogino, Atsugi-City, Kanagawa, Japan; 2 Waseda University, 3-4-1 Okubo, Shinjuku-ku, Tokyo, Japan; 3 Oracle Corporation Japan, SBS Tower 4-10-1, Yoga, Setagaya-ku, Tokyo, Japan; 4 Tokyo Woman’s Christian University, 2-6-1 Zempukuji, Suginami-ku, Tokyo, Japan

**Keywords:** Web page link, Link classification, Accessibility

## Abstract

Web pages are used for a variety of purposes. End users must understand dynamically changing content and sequentially follow page links to find desired material, requiring significant time and effort. However, for visually impaired users using screen readers, it can be difficult to find links to web pages when link text and alternative text descriptions are inappropriate. Our method supports the discovery of content by analyzing 8 categories of link types, and allows visually impaired users to be aware of the content represented by links in advance. This facilitates end users access to necessary information on web pages. Our method of classifying web page links is therefore effective as a means of evaluating accessibility.

## Introduction

Web pages are used for a variety of purposes, including electronic commerce and e-learning. Because web pages are used for a wide array of purposes, many types of content can be included in one page. In addition, in many cases the reloading of a page can cause its content to change. For example, some web pages include many advertisements, or as in the case of Twitter, real-time updates. Many pages also use dynamic content such as Flash and JavaScript. When end users access these pages, they must be able to interpret this changing content by following the appropriate links. End users who are disabled or elderly, however, may have difficulty utilizing web pages that change frequently. Therefore, it is important to end users that web page designers improve operability.

Web accessibility, which refers to web pages being easily usable by all end users, is also regarded as important. There are many guidelines pertaining to accessibility (Section 508 Homepage 2011; Web Content Accessibility Guidelines (WCAG) 2008). In particular, visually impaired users often use support software such as screen readers (Freedom Scientific Inc. 2011). Screen readers are software programs that read aloud the material displayed on screens.

However, the current degree of support for visually impaired users is inadequate. When end users want to find web pages, they must often follow a number of links. It is sometimes difficult for visually impaired users using screen readers to find web page links. Screen readers usually read both link text and the alternative text associated with images. It is difficult for visually impaired users to locate a link when the link text or alternative text descriptions are not appropriate. It is preferable for visually impaired users to know the type of content associated with a link before they actually follow or click on it. For example, links to advertisements may redirect the user to other sites. Visually impaired users cannot know that they have navigated to another site until the screen reader begins to read the content aloud. There are tools and methods to identify problems faced by visually impaired users when they access web pages. However, the purpose of these methods is to reveal the problems to web page designers, and thus it is necessary for end users to wait until the pages are finally modified.

Therefore, we propose a method of automatically distinguishing categories of links on web pages. Web pages are analyzed by extracting links from the pages’ HTML sources. Visually impaired users can be aware of the content represented by each link beforehand, and end users can minimize the time spent following unnecessary links.

## Web accessibility

In the context of IT (Information Technology), accessibility refers to the degree to which services or software are easily usable, particularly by the elderly and disabled. The accessibility of web pages is called “web accessibility”.

### Definition of web accessibility

Web accessibility refers to construction of a web site such that all users can access its information, regardless of their age or physical limitations, and can easily navigate its environment. Visually impaired users often use screen readers. A screen reader is an application that converts onscreen text into speech. When this type of software reads text containing a web site link, it generally reads both the text and the link. In addition, hardware is available that displays onscreen information as braille. It is imperative that web designers produce web pages that effectively support the use of these tools.

### Web accessibility guidelines

A variety of guidelines pertaining to web accessibility have been prepared. Two well-known guidelines are the WCAG 2.0 and the United States government Section 508 Amendment to the Rehabilitation Act.

The Web Content Accessibility Guidelines 2.0 (WCAG 2.0) provides recommendations regarding the accessibility of web content. It was established by the World Wide Web Consortium (W3C) and was written for all web designers, web site creators, and authoring tool developers. Web content developed in conformity with these guidelines does not benefit only impaired persons; regardless of the device used, such as cell phone, PC browser, smartphone, and so on, it provides standards for making information on web pages easy to find for all end users. The Section 508 Amendment to the Rehabilitation Act is a law requiring that all IT devices, software, and web sites procured, developed, or used by United States government agencies must be accessible to those with disabilities. As a result, all companies that deliver products for use by public institutions and the United States government must place some emphasis on web accessibility.

## Related works

Several methods can be used to identify problems encountered by visually impaired users as they interact with web pages. G. Gay et al. proposed a method to discern accessibility problems that cannot be identified automatically by accessibility checking tools (Greg and Cindy 2010). For example, when a web page contains movie content, this tool recognizes that the page may have accessibility issues. A. Gonzales et al. defined a platform-independent accessibility API framework (Gonzalez and Reid 2005). This method can be used by web designers to identify and address accessibility problems. With the method presented in this paper, visually impaired users can be aware of the content of linked web pages without actually accessing the links. Even if web site accessibility is insufficiently implemented, our approach makes page contents intelligible to users.

## Web page link analysis

Our method allows visually impaired users to locate and identify content using web page links. We analyze links in web pages and classify linked components, and the type of linked page is shown to end users.

### Web page links for visually impaired users

When end users find a desired web page, they must often follow one or more links. When visually impaired users find a desired link, they use screen readers. However, end users must distinguish the desired link from among many other links on the same page. In cases where a web designer gives priority to visual design, or if the structure of a web page is not appropriately defined, many links are not read aloud in the proper order. Screen readers can read links’ text content as well as images alternative text descriptions. However, when this information is inappropriate, visually impaired users may have difficulty understanding linked content and may therefore struggle to find a desired link. When visually impaired users follow a link, they must wait until the screen reader parses the page contents before they can understand the content of the linked page. These users cannot identify inappropriate or unwanted links, such as those associated with advertisements, until the screen reader has at least partially evaluated the page. In addition, many pages use dynamic content such as Flash, which requires the use of plug-in software that makes it difficult for visually impaired users to access content due to screen reader incompatibility.

### Categorization of web page contents

Our research supports end users by categorizing the content of web page links. To achieve this, we surveyed the general content contained in 2,541 randomly selected web pages. Link classification results are shown in Table 1.

**Table 1:** **Results of link classification for 2,541 web pages**

Categoly	Number of pages
1. Article	1,314
2. Image	55
3. File	79
4. Plug-in	68
5. Input format	194
6. Other site	546
7. Link page	171
8. Link to own page	114

We then classified the links that led to these pages into 8 categories, as shown below.

Article: Main content consisting of sentences, such as news pages
Image: Image files, such as photos and pictures
File: Various file types, such as movies and PDF format
Plug-in: Web pages containing plug-ins, such as Adobe Flash format
Input form: Web pages with input forms, such as login forms or address entry forms
Other site: Web pages located at a different domain
Linnk page: Web pages containing many links to other pages
Link to own page: Any web pages containing links to locations within the same page

These categories indicate to visually impaired users the behavior of links on web pages they are visiting. When end users follow a link in order to obtain information on the web site, they understand that they should use links of the “Article” category. Links in the “Link to own page” category do not transfer the user to a different web page, but rather move to the location on the current page that contains the desired content. When end users wish to enter data, the appropriate link would belong to the “Input form” category. With the link categories “Image”, “File”, and “Plug-in”, accessibility is often insufficient and visually impaired users generally know that linked information may not be obtained beforehand. Links in the “Other site” category lead to destinations not on the current web page.

Therefore when end users believe that the information they are looking for exists on the current web site, they can assume that links of the other site type are not the correct ones. In addition, since the content of external web pages can change frequently, end users can prevent confusion by not following this type of link unnecessarily.

## Method of link classification

In our method, the classification of web page links is based on URL and HTML source code. This classification is performed using the following strategy:

1. Classifying by a links URL
  This strategy is used to categorize “Image”, “File” “Other site” and “Link to own page”.
2. Classifying by analysis of a web pages HTML source code
  This strategy is used to categorize “Article”, “Plug-in”, “Input format” and “Link page”.

### Classifying by a link’s URL

In this strategy, links are categorized based on the file type extension used in the link fs URL. These link sf URLs are categorized in the following order:

1. Image contents are categorized. When a links content is of the “Image” type, the end of the URL uses an image file extension. We defined “jpg”, “gif”, “png”, “jpeg”, “tif”, “tiff”, “bmp” and “ico” as the extensions of image files.
2. File contents are categorized. When a linkfs content is of the “File” type, the end of the URL consists of a string other than “htm” or “html”.
3. “Other site” contents are categorized. When the domain name of a links URL is different from that of the target page, this link is categorized as “Other site”.
4. “Link in own page” contents are categorized. Applicable when the “#” character and keyword string are added after the file name of the URL.

**Figure 1:**
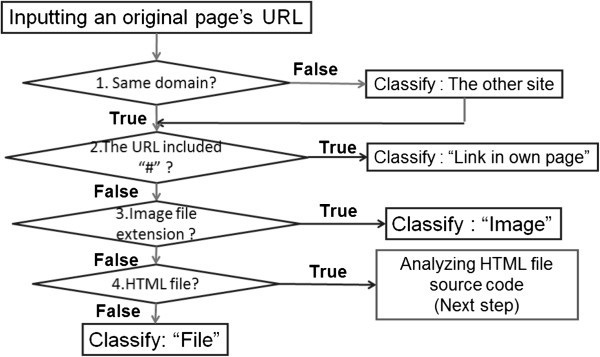
**Classification analysis flow by links URL.**

The flow of this analysis is shown in Figure 1.

### Classifying by analysis of a linked web page’s HTML source code

In this strategy, links are categorized based on the linked web pagefs HTML source code. Classification of an “Article” is based on the length of a sentence surrounded by various tags, such as div tag, td tag, li tag, or ol tag. Our prior survey showed that an “Article” web page is comprised of more than 100 Japanese characters. When the number of Japanese characters is more than 100, web pages are categorized as containing “Article” contents. When the Object tag is included in a web page, the page is categorized as containing “Plug-in” contents. In many cases, the “Plug-in” contents use Adobe Flash. When more than 2 input tags such as input tag, select tag, Form tag, and textarea tag are included in a web page, the page is categorized as containing “Input form” contents. The classification of “Link page” is based on the number of anchor tag in the web page. When there are more than 10 links in a page, it is categorized as containing “Link page” contents.

**Figure 2:**
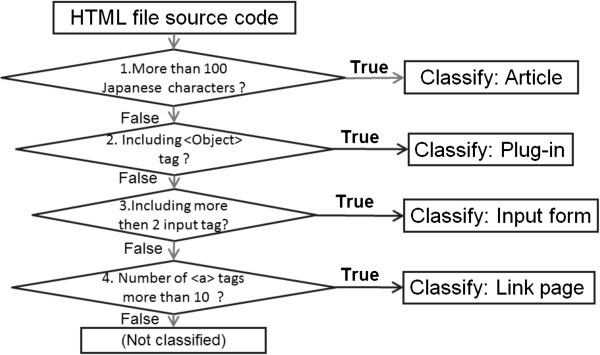
**Classification analysis flow by a HTML source analysis.**

The flow of this analysis is shown in Figure 2.

### Overlap and priority of the link category

With the above strategies, a web page may be assigned to two or more categories. For example, when a web page has more than 100 Japanese characters and more than 10 anchor tags, this page is classified into both the “Article” and “Link page” categories. It is necessary that end users are clearly presented with the category of the web page’s contents, so we assign priorities to page categories. The “Other site” category is not directly related to the contents of the web page. For example, when a web page is categorized as both “Article” and “Other site”, End users can clearly identify that the page is classified as an “Article”, but with content residing on an “Other site”. We assign priorities to web page classifications based on the importance to end users when they are reading pages, as follows:

1. Image and File
  The “Image” and “File” categories do not overlap with the “Other site” category. Highest priority is assigned to the “Image” and “File” categories.
2. Link in own page
  The “Link in own page” category is assigned for the purpose of directly jumping to content that end users want to find. Second priority is assigned to the “Link in own page” category.
3. Input form
  The “Input form” category applies to a web page that requires end users input, such as login ID, password, or e-mail address. It is necessary to alert end users to items required on a registered users web page. Third priority is assigned to the “Input form” category.
4. Plug-in
  The “Plug-in” category indicates a web page that requires end users to install a plug-in module before page contents can be accessed. We alert end users to this fact by assigning the fourth priority to the “Plug-in” category.
5. Article
  The “Article” category is a classification of general web pages with heavy text content. Fifth priority is assigned to the “Article” category.
6. Link page
  A large number of anchor tags are often included in web pages. The lowest priority is assigned to the “Link page” category.

## Evaluation

We evaluated the effectiveness of our web pages links classification method. We assumed that the web site accessibility ranking of various companies by Nikkei was accurate (Nikkei 2010). Visually impaired users often use screen readers. While these programs are able to read text on screens, they cannot read image files or plug-in pages. We compared the web pages of 2 companies with a significant difference in accessibility scores. The score of Company A was 22.0 points, while that of Company B was 17.8 points. We evaluated the web sites of these 2 companies using our method as detailed above. The web site of Company A contained 11,332 web pages, while that of Company B contained 14,687 web pages. The results of the web page classification using our method are shown in Table 2.

**Table 2:** **Classification of 2 companies web pages links using our method**

Categoly	Company A (11,332)	Company B (14,687)
Article	73.4% (8,221)	62.9% (9,245)
Image	0% (0)	0.04% (7)
File	2.4% (276)	5.0% (737)
Plug-in	0.1% (15)	1.2% (171)
Input form	2.9% (330)	2.2% (322)
Link page	0.5% (53)	1.8% (261)
Link to own page	17.1% (1,939)	22.2% (3,259)
Not classified	4.3% (498)	4.7% (685)
(Other site) ^∗^	(27.3%) (3,089)	(14.0%) (2,053)

^∗^The “Other site” category can overlap other categories.

The “Article” category consists of text. These pages can therefore be read aloud by screen readers, and visually impaired users can understand the content. The web site of Company B contained more than 2 times the number of links of the “File” type compared to Company A. These pages are less accessible to visually impaired users. For example, the contents of movie files that do not contain voice information cannot be understood. Even if sounds and voices are included in a movie file, support for visually impaired users is not enough. When visually impaired users understand movie file contents, movie files should include explanation of the movie with voices. We evaluated a page as having high accessibility if it had few file-type links. The web site of Company B contained more than 10 times the number of pages in the “Plug-in” category compared to Company A. We cannot know whether a plug-in module is accessible to end users with impairments of various kinds, and we therefore evaluated a site as having high accessibility if it had few “Plug-in” category pages.

Our results confirmed the findings of Nikkei, namely that the web site of Company A demonstrated superior accessibility. Our method of classifying a web page’s links is therefore effective as a means of evaluating accessibility.

## Conclusion

In this paper, we proposed a method of classifying web pages using link analysis. We classified links into categories, and used these categories to successfully assess web page accessibility.

Future works are as follows:

Finding out category classification
Our survey classified web pages into 8 categories. To more appropriately perform instruction-linked page for end users, we will attempt to determine web page categories.
Developing highly accurate classification
We will consider ways in which details of web page classification, such as HTML tags, contribute to accessibility support.
Supporting style sheet analysis
Expression of links varies when style sheets are used. We will evaluate the importance of links based on the method with which they are expressed.
